# Can mobilization of bone marrow stem cells be an alternative regenerative therapy to stem cell injection in a rat model of chronic kidney disease?

**DOI:** 10.14814/phy2.15448

**Published:** 2022-09-06

**Authors:** Shereen Morsy, Mona F. Mansour, Mohamed Abdo, Yasser El‐Wazir

**Affiliations:** ^1^ Physiology Department, Faculty of Medicine Suez Canal University Ismailia Egypt; ^2^ Centre of Excellence in Molecular and Cellular Medicine, Faculty of Medicine Suez Canal University Ismailia Egypt

**Keywords:** adipose tissue‐derived mesenchymal stem cells, adriamycin, CD34^+^ stem cells, chronic kidney disease, granulocyte colony‐stimulating factor, malondialdehyde

## Abstract

Chronic kidney disease (CKD) is a priority health problem affecting 36% of Egyptians. Adipose‐derived mesenchymal stem cells (ADMSCs) have multidifferentiation capacity and the ability to restore several types of cells including damaged renal cells. Granulocyte colony‐stimulating factor (G‐CSF) is known to mobilize hematopoietic stem cells from bone marrow to the peripheral circulation. The aim of this study was to compare the effect of endogenous CD34^+^ cells mobilization and exogenous ADMSCs administration in the treatment of a rat model of adriamycin (ADR)‐induced CKD. A total of 48 male albino rats of the local strain (200 ± 50 g) were equally divided into four groups: control negative, ADR (control positive), ADMSCs group, and G‐CSF group. Six rats from each group were sacrificed after 4 weeks and the other 6 after 12 weeks. Renal function was assessed frequently by measuring serum creatinine, albumin, urea, 24‐h urinary protein level, and hemoglobin level throughout the study. Oxidative stress markers malondialdehyde (MDA) and total antioxidant (TAO) were measured on day 28. CD‐34^+^ cell percentage was measured on day 9. After the sacrification of the rats, kidneys were removed for histopathological assessment. Results revealed that both ADMSCs and G‐CSF significantly improved serum creatinine, albumin, urea, 24‐h urinary protein level, and histopathological damage score, with the G‐CSF‐treated group showing better improvement in 24‐h urinary protein level, serum albumin, and histopathological damage score compared with ADMSCs‐treated group. The G‐CSF group also had significantly higher levels of CD34^+^ cells. Oxidative stress markers (MDA and TAO) levels were significantly improved with both therapies. We conclude that mobilization of endogenous hematopoietic stem cells by G‐CSF is more effective than exogenously injected ADMSCs in protecting the kidneys against AD‐induced toxicity.

## Graphical abstract

Chronic kidney disease (CKD) is a priority health problem affecting 36% of Egyptians. Regenerative therapy can provide hope of restoring the normal life of those patients. In this paper, we show that mobilization of endogenous hematopoietic stem cells by granulocyte colony‐stimulating factor is at least as effective as exogenously injected adipose‐derived mesenchymal stem cells in protecting the kidneys in a rat model of CKD induced by adriamycin toxicity.

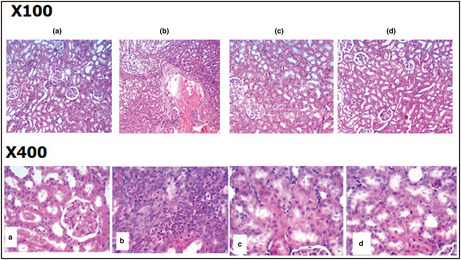

## 1 INTRODUCTION

Chronic kidney disease (CKD) is considered a priority health problem affecting 14% of the world population and 36% of Egyptians (Bogdan et al., 2016), causing substantial global morbidity as well as increased cardiovascular and all‐cause mortality (Shlipak et al., 2021).

Patients on renal dialysis report a reduced quality of life, because although dialysis replaces kidney filtration function, other kidney functions, such as EPO production and Vitamin D activation, are not restored (Eirin & Lerman, 2014). Despite advances in renal transplantation, long transplant waiting lists are major concerns for nephrologists and patients alike (Bussolati et al., 2009).

Adipose tissue‐derived mesenchymal stem cells (ADMSCs) injection has been shown to be relatively safe and potentially beneficial to some CKD patients already on standard medical treatment (Villanueva et al., 2019) but with concerns about the safety of grafting. Some studies have demonstrated that ADMSCs can reduce the severity of the ischemic renal injury and prevent the progression of subsequent renal fibrosis by suppressing oxidative stress and inflammatory response (Chen et al., 2011). However, there are several issues that hinder adopting ADMSCs as a standard line of treatment in CKD, most importantly the need for immunosuppressive therapy in allogeneic cell transplantation.

Granulocyte colony‐stimulating factor (G‐CSF), which is known to mobilize HSCs from the bone marrow (BM) to the peripheral circulation, may provide a more appropriate alternative form of cell therapy over infused MSCs. The protective action of G‐CSF on renal function has been demonstrated in mouse models of acute renal failure, although there has been little investigation on its role in CKD (Andrade et al., 2013). Furthermore, G‐CSF promotes postnatal renal repair by recruiting and influencing macrophages toward a reparative state. G‐CSF induces renal repair through tubular epithelial cell replacement and attenuation of interstitial fibrosis and is vital to kidney growth and the promotion of endogenous repair and resolution of inflammatory injury (Yan et al., 2020).

In the current study, we compare the regenerative capacity of ADMSCs and G‐CSF in adriamycin‐induced chronic nephropathy in rats. In addition, modulating oxidative stress and apoptosis as underlying mechanisms in mediating tissue regeneration were assessed in the two treatment modalities.

## 2 MATERIALS AND METHODS

This randomized experimental interventional study was carried out in the animal house in the Physiology Department, the laboratories of physiology and clinical pathology, and in the tissue culture unit, Center of Excellence, Faculty of Medicine, Suez Canal University. Forty‐eight male albino rats of local strain with an average weight of (200 ± 50 g) were bought from the National Research Center in Cairo to be included in this study. Animals were housed in the Animal House, Faculty of Medicine, Suez Canal University in spacious plastic cages at a controlled room temperature and were kept with free access to a standard rat chow diet and tap water. They were left for 1 week for acclimatization before the start of the study. Handling and care of animals before and during the experimental procedures were done in accordance with the guidelines of the Animal Ethical Committee, Faculty of Medicine, Suez Canal University, which approved the study.

### 2.1 Experimental groups

Rats were randomly divided into four equal main groups with 12 rats in each. In Group I (control group), the rats received a single intravenous injection (tail vein) of 0.5 ml saline. In Group II (Adriamycin group), the rats received a single intravenous injection (tail vein) of Adriamycin at a dose of 5 mg/kg of body weight (Montilla et al., 1997) and 1 ml saline 1 week after adriamycin injection. In Group III (adipose‐derived stem cells group), rats received ADMSCs (2 × 10^6^ cells suspended in 1 ml saline) 1 week after the Adriamycin injection (Zickri et al., 2012). In Group IV (G‐CSF group), the rats received Adriamycin by the same route and the same dose as Group II, and then G‐CSF was given 2 h after the Adriamycin injection. G‐CSF was subcutaneously injected daily for five consecutive days at a dose of 70 μg/kg diluted in 0.5 ml glucose 5% (Sadek et al., 2016). Each group was subdivided into two halves: six of the rats were sacrificed after 4 weeks, whereas the other six were sacrificed after 12 weeks.

### 2.2 Induction of chronic kidney disease (CKD)

Nephropathy was induced by a single intravenous (IV) injection of Adriamycin (Doxorubicin hydrochloride [50 mg/25 ml saline], EIMC United Pharmaceuticals, EUP, Cairo, Egypt), at a dose of 5 mg/kg of body weight (Zickri et al., 2012).

### 2.3 Collection and preparation of ADMSCs

The process of separation of adipose‐derived stem cells (ADMSCs) included the following two major steps: (a) isolation and (b) digestion of adipose tissue (AT).

#### 2.3.1 Isolation of adipose tissue

AT was taken from apparently healthy rats' peritoneum after sacrification and collected in a sterile bottle containing an equal volume of phosphate‐buffered saline (PBS) (Lonza‐Belgium, cat. no. BE17‐516F) with 1%–5% Streptomycin, penicillin (p/s) (cat. no. P4333, Sigma‐Aldrich). The bottle was vigorously shaken for 5–10 s and then allowed to settle. The supernatant (containing blood and PBS) was removed and then repeatedly washed 2–3 times until the supernatant became clear. The AT was transferred to a Petri dish and minced using scissors and a scalpel into small parts to ease digestion.

#### 2.3.2 Digestion with collagenase

About 2 ml Collagenase type I (cat. no. C‐0130, Sigma‐Aldrich), prepared at 1–2 mg/ml in TESCA buffer (containing 50 mM TES, 0.36 mM calcium chloride, pH 7.4) at 37°C was added to a sterile Petri dish containing minced AT. The mixture was left for 30 min at 37°C and 5% CO_2_ with frequent shaking until the mixture acquired a thick consistency.

The action of collagenase was neutralized by adding Dulbecco's modified Eagle's medium (DMEM) low glucose, L‐glutamine, and sodium pyruvate (Biowest, cat. no. L0060500), containing 10% heat‐inactivated fetal bovine serum (FBS, cat. no. F6178, Sigma‐Aldrich). The sample was pipetted several times to aid disintegration then transferred to 15‐ml sterile Falcon tubes (large pieces were discarded). Centrifugation at 2000 rpm for 5 min was done to obtain the stromal vascular fraction (containing adipose stem cells). The pellet was resuspended in sterile distilled water to lyse red blood cells and incubated in ice for 10 min. When the pellet became white, the supernatant was aspirated and resuspended in a control medium: DMEM (low glucose), FBS 15%, and 1% antibiotic. The suspension was transferred to a culture flask and incubated at 37°C and 5% Co_2_ for 72 h (Lopez & Spencer, 2011)

### 2.4 Labeling the ADMSCs

Before cell transplantation, the cultured cells were impregnated with an injectable solution of ferumoxides 25 μg Fe/ml (feridex) (Bayer Health Care Pharmaceuticals) for 24 h with 375 ng/ml poly L‐lysine added 1 h before cell incubation. Feridex‐labeled MSCs were washed in PBS, trypsinized using Trypsin (Lonza, Walkersville, cat. No. D2287), washed, and resuspended in 0.01 mol/L PBS at a concentration of 1 × 1,000,000 cells/ml. Labeling was histologically assessed using Prussian blue (Sarhan et al., 2014).

### 2.5 Administration of ADMSCs

The intravenous route for administration of ADMSCs was chosen based on previous studies that showed significant engraftment of MSCs in injured tissue after IV injection of MSCs (Ezquer et al., 2008). Moreover, previous studies have reported that administration of MSCs into the renal artery could be associated with two major complications: (*i*) renal infarcts and loss of function and (*ii*) ectopic differentiation into adipocytes within the glomeruli (Zoja et al., 2012).

### 2.6 Collection and preparation of mononuclear cells CD34+ cells

Collection of blood samples was done 4 days following G‐CSF (Neupogen) provided in the form of prefilled syringes of 0.5 ml containing 30 million units (300 μg) of filgrastim (recombinant methionyl human G‐CSF, r‐metHuG‐CSF, from *Escherichia coli* K12) from the Oncology Unit, Suez Canal University Hospital (Eidenschink et al., 2012).

#### 2.6.1 Extraction of PBMCs

Density gradient centrifugation was performed to isolate the mononuclear cells from peripheral blood (PB) (Fleury et al., 2009).

#### 2.6.2 Detection of PB CD34+ cells

Flow cytometric assessment was done on a flow cytometry system (The BD FACSLyricTM Flow Cytometer, BD Life Sciences), using fluorescein isothiocyanate (FITC)‐CD34+ monoclonal antibody (bs‐0646R‐FITC; Beijing Bioss Biotechnology Co., Ltd.). We used only CD34 FITC to assess CD34 expression in the studied group. For each sample, two tubes were used: the first unstained tube was used to decide the separation point between negative and positive populations, and the second tube contained a FITC‐labeled monoclonal antibody against CD34.

### 2.7 Follow‐up and monitoring

**FIGURE 1:**
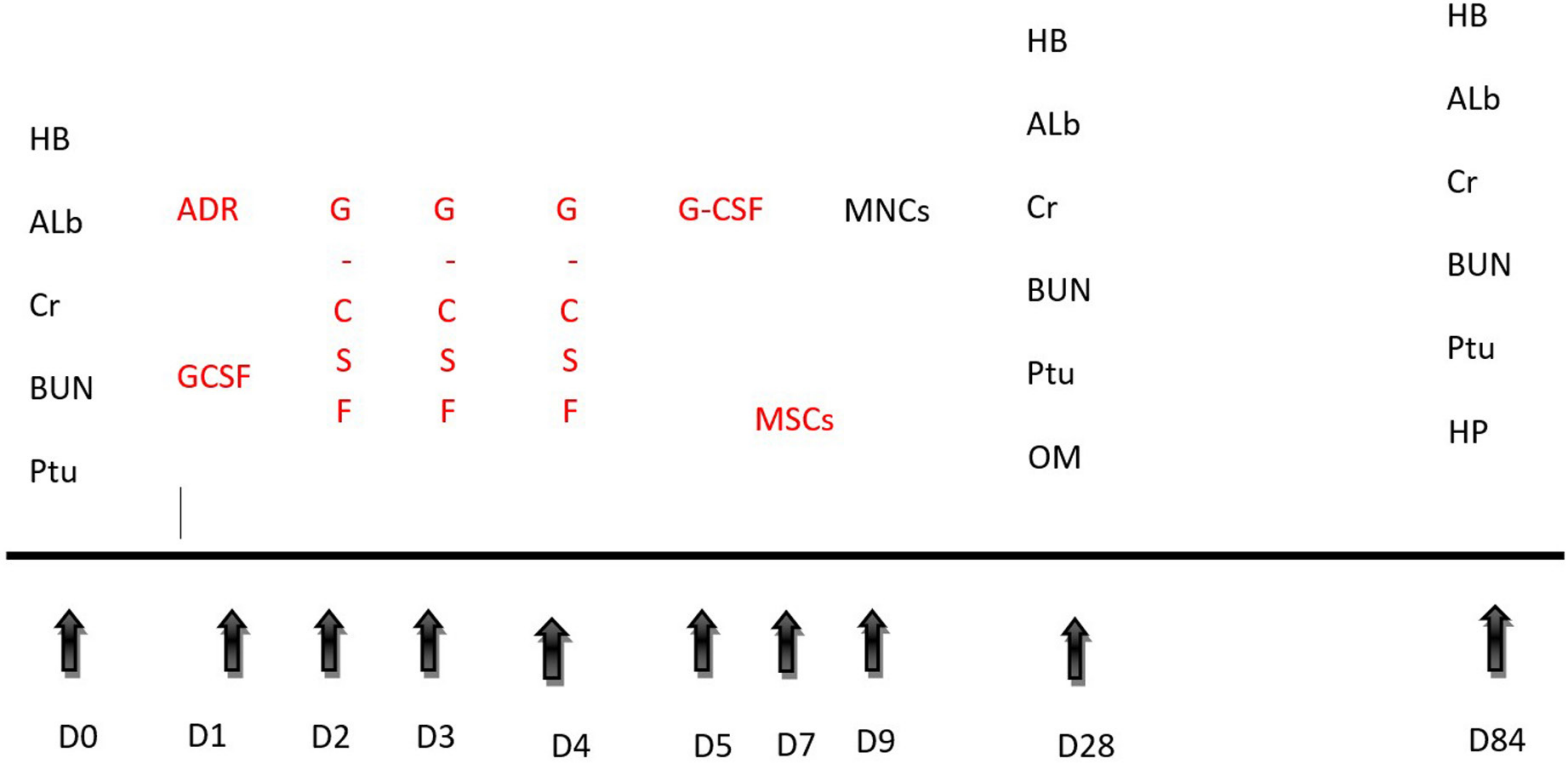
Time schedule for different workflow steps. Administered chemicals or cells for intervention are colored in red color, while measured parameters are colored in black. ADR, adriamycin; Alb, albumin; BUN, blood urea nitrogen; Cr, creatine; G‐CSF, G colony‐stimulating factor; HB, hemoglobin; HP, histopathology; MNCs, counting mononuclear cells; MSCs, AD‐MSCs injection; OM, oxidative markers; Ptu, proteinuria.

The study follow‐up schedule was summarized in Figure 1, including:

1. Collection of retro‐orbital blood samples for assessment of renal function tests.
2. Urine samples: metabolic cages were used for the collection of urine samples. Proteinuria was determined using an automatic biochemistry analyzer (DADE Xpand, USA).
3. Measurement of the TAO in homogenized renal tissue by the colorimetric method, using TAC Assay Kit (cat. no. BYEK2200, Biopes).
4. Assessment of the MDA in homogenized renal tissue using MDA ELISA Kit (cat. no.: BYEK1371, Biopes).
5. Histopathological examination of renal tissue.

### 2.8 Serum albumin creatinine, blood urea nitrogen measurement

They were assessed for all groups at Day 0 and week 11 + 6 days by the end of the study. A set of blood tubes without anticoagulant were centrifuged at 3000 *g* for 15 min and stored at −20°C for assessment of serum creatinine (Creatinine Assay Kit, cat. no. MAK080, Sigma‐Aldrich) and BUN (Urea Nitrogen [BUN] Colorimetric Detection Kit, cat. no. EIABUN, Invitrogen) by Siemens Advia 1800 Chemistry Analyzer System (Qin et al., 2013).

### 2.9 Data analysis

All numerical data were expressed as mean ± *SD* and were analyzed with SPSS statistical software version 20. One‐way analysis of variance (ANOVA) was used to compare the means of the three study groups and to determine the presence of a significant difference among the groups. Two methods of ANOVA were used (multivariate ANOVA and repeated measures ANOVA). If there was a significant difference between groups, a post hoc test was used to test the difference between each pair of means. Chi‐square was used to compare histopathological scoring in the three groups and to determine the significance between groups. A *p* value of <0.05 was considered statistically significant.

## 3 RESULTS

### 3.1 Assessment of renal function

#### 3.1.1 Twenty‐four‐hour total protein level in urine

As shown in Table 1, the 24‐h protein urine level on day 5 was significantly higher on induction of CKD compared with normal rats (*p* = 0.000). However, on both day 28 and day 84, therapy groups III and IV showed significant improvement in their 24‐h protein urine level compared with group II. Group IV returned to their normal 24‐h protein urine level, as evidenced by an insignificant difference compared with Group I.

**TABLE 1:** Twenty‐four hours of urinary protein levels in all study groups throughout the study

Title	Group I control negative	Group II control positive	Group III ADMSCs	Group IV G‐CSF	*p* value#
Proteinuria day 0 mg/24 h	668.20 ±51.36	665.90 ±37.54	656.60 ±40.19	667.50 ±45.83	0.929
Proteinuria day 5 mg/24 h	643.70 ±44.48	2381.40 ±240.65 ^a^	2354.90 ±171.85 ^a^	2449.50 ±257.83^a^	0.000*
Proteinuria day 28 mg/24 h	660.00 ±61.51	2848.30 ±585.88^a^ ^,^ ^c^ ^,^ ^d^	1449.00 ±282.25^a^ ^,^ ^b^ ^,^ ^d^	944.20 ±27.84^a^ ^,^ ^b^ ^,^ ^c^	0.000*
Proteinuria day 84 mg/24 h	653.80 ±36.29	2841.20 ±461.85^a^ ^,^ ^c^ ^,^ ^d^	982.40 ±77.85^a^ ^,^ ^b^	815.80 ±81.11^b^	0.000*
*p*‐value	0.272	0.015**	0.002**	0.002**	

Abbreviations: ADSC, adipose tissue‐derived mesenchymal stem cells; ANOVA, analysis of variance; G‐CSF, Granulocyte colony‐stimulating factor.

^a^ *p* value <0.05 compared with group I using post hoc Tukey test.

^b^ *p* value <0.05 compared with group II using post hoc Tukey test.

^c^ *p* value <0.05 compared with group III using post hoc Tukey test.

^d^ *p* value <0.05 compared with group IV using post hoc Tukey test.

* Statistically significant *p* value <0.05 level, using multivariate ANOVA test

** Statistically significant *p* value <0.05 level, using repeated measure ANOVA test.

#### 3.1.2 Serum albumin level

As shown in Table 2, the serum albumin level on day 5 was significantly lower on induction of CKD compared with normal rats (*p* = 0.000). However, on both day 28 and day 84, therapy groups (III and IV) showed significant improvement in their serum albumin level compared with group II (*p* = 0.000). On day 84, both therapy groups (III and IV) returned to their normal serum albumin level, as evidenced by insignificant difference compared with group I.

**TABLE 2:** Comparison of serum albumin levels (g/dl) throughout the study among study groups

Title	Group I control negative	Group II control positive	Group III ADMSCs	Group IV G‐CSF	*p* value#
	Mean ± *SD*	Mean ± *SD*	Mean ± *SD*	Mean ± *SD*	
Day 0	3.93 ± 0.40	4.16 ± 0.30	3.97 ± 0.34	3.81 ± 0.37	0.191
Day 5	3.97 ± 0.33	3.55 ± 0.25^a^	3.55 ± 0.27^a^	3.69 ± 0.19^a^	0.003*
Day 28	4.06 ± 0.37	2.30 ± 0.22^a^ ^,^ ^c^ ^,^ ^d^	3.57 ± 0.22^a^ ^,^ ^b^	3.44 ± 0.30^a^ ^,^ ^b^	0.000*
Day 84	4.10 ± 0.29	2.20 ± 0.20^a^ ^,^ ^c^ ^,^ ^d^	3.84 ± 0.25^b^	3.98 ± 0.29^b^	0.000*
*p*‐value	0.336	0.004**	0.02**	0.029**	

Abbreviations: ADSC, adipose tissue‐derived mesenchymal stem cells; ANOVA, analysis of variance; G‐CSF, Granulocyte colony‐stimulating factor.

^a^ *p* value <0.05 compared with group I using post hoc Tukey test.

^b^ *p* value <0.05 compared with group II using post hoc Tukey test.

^c^ *p* value <0.05 compared with group III using post hoc Tukey test.

^d^ *p* value <0.05 compared with group IV using post hoc Tukey test.

* Statistically significant *p* value <0.05 level, using multivariate ANOVA test

** Statistically significant *p* value <0.05 level, using repeated measure ANOVA test.

#### 3.1.3 Serum creatinine

As shown in Table 3, on day 5, the creatinine levels were significantly higher on induction of CKD compared with normal rats (*p* = 0.000). However, on both day 28 and day 84, therapy groups (III and IV) showed significant improvement in their creatinine levels compared with group II.

**TABLE 3:** Comparison of serum creatinine level (mg/dl) throughout the study among study groups

	Group I control negative	Group II control positive	Group III ADMSCs	Group IV G‐CSF	*p* value*
Day 0	0.20 ± 0.07	0.18 ± 0.06	0.19 ± 0.06	0.18 ± 0.06	0.872
Day 5	0.18 ± 0.04	0.29 ± 0.06^a^	0.29 ± 0.06^a^	0.31 ± 0.06^a^	0.003*
Day 28	0.25 ± 0.05	0.65 ± 0.11^a^ ^,^ ^c^ ^,^ ^d^	0.54 ± 0.05^a^ ^,^ ^b^	0.57 ± 0.07^a^ ^,^ ^b^	0.000*
Day 84	0.24 ± 0.05	0.74 ± 0.09^a^ ^,^ ^c^ ^,^ ^d^	0.48 ± 0.08^a^ ^,^ ^b^	0.48 ± 0.08^a^ ^,^ ^b^	0.000*
*p*‐value	0.133	0.003**	0.003**	0.003**	

Abbreviations: ADSC, adipose tissue‐derived mesenchymal stem cells; ANOVA, analysis of variance; G‐CSF, Granulocyte colony‐stimulating factor.

^a^ *p* value <0.05 compared with group I using post hoc Tukey test.

^b^ *p* value <0.05 compared with group II using post hoc Tukey test.

^c^ *p* value <0.05 compared with group III using post hoc Tukey test.

^d^ *p* value <0.05 compared with group IV using post hoc Tukey test.

* Statistically significant *p* value <0.05 level, using multivariate ANOVA test

** Statistically significant *p* value <0.05 level, using repeated measure ANOVA test.

#### 3.1.4 Serum urea level

As shown in Table 4, on day 0 and day 5, there was no statistically significant difference among all study groups (*p* = 0.859 and *p* 0.854, respectively). On both day 28 and day 84, therapy groups (III and IV) had significantly lower serum urea levels compared with group II (*p* = 0.000).

**TABLE 4:** Comparison of serum urea levels (mg/dl) at different days between different study groups

Title	Group I control negative	Group II control positive	Group III ADMSCs	Group IV G‐CSF	*p* value*
Day 0	26.40 ± 3.84	26.00 ± 3.02	27.20 ± 3.05	26.90 ± 3.41	0.859
Day 5	26.30 ± 3.65	27.40 ± 4.84	27.10 ± 3.96	25.90 ± 4.68	0.854
Day 28	27.30 ± 3.13	69.50 ± 5.99^a^ ^,^ ^c^ ^,^ ^d^	48.40 ± 3.5^a^ ^,^ ^b^	49.20 ± 3.46^a^ ^,^ ^b^	0.000*
Day 84	26.80 ± 2.39	80.40 ± 6.11^a^ ^,^ ^c^ ^,^ ^d^	38.40 ± 4.10^a^ ^,^ ^b^	41.00 ± 6.04^a^ ^,^ ^b^	0.000*
*p* value	0.48	0.003**	0.006**	0.006**	

Abbreviations: ADSC, adipose tissue‐derived mesenchymal stem cells; ANOVA, analysis of variance; G‐CSF, Granulocyte colony‐stimulating factor.

^a^ *p* value <0.05 compared with group I using post hoc Tukey test.

^b^ *p* value <0.05 compared with group II using post hoc Tukey test.

^c^ *p* value <0.05 compared with group III using post hoc Tukey test.

^d^ *p* value <0.05 compared with group IV uing post hoc Tukey test.

* Statistically significant *p* value <0.05 level, using multivariate ANOVA test

** Statistically significant *p* value <0.05 level, using repeated measure ANOVA test.

#### 3.1.5 Hemoglobin content

As shown in Table 5, on day 0, there was no statistically significant difference among all study groups (*p* = 0.962). On day 84, mean hemoglobin for group II showed a statistically significant difference compared with both groups I and III.

**TABLE 5:** Comparison between Hemoglobin levels (gm/dl) among all study groups

Title	Group I control negative	Group II control positive	Group III ADMSCs	Group IV G‐CSF	*p* value#
Day 0	14.11 ± 1.15	14.25 ± 1.06	14.36 ± 1.40	14.13 ± 1.13	0.962
Day 84	14.50 ± 1.00	11.50 ± 0.5^a^ ^,^ ^c^	14.10 ± 0.55^b^	14.90 ± 0.74	0.000*

Abbreviations: ADSC, adipose tissue‐derived mesenchymal stem cells; ANOVA, analysis of variance; G‐CSF, Granulocyte colony‐stimulating factor.

^a^ *p* value <0.05 compared with group I using post hoc Tukey test.

^b^ *p* value <0.05 compared with group II using post hoc Tukey test.

^c^ *p* value <0.05 compared with group III using post hoc Tukey test.

* Statistically significant *p* value <0.05 level, # ANOVA test.

#### 3.1.6 Histopathological examination of renal tissue using H&E

**FIGURE 2:**
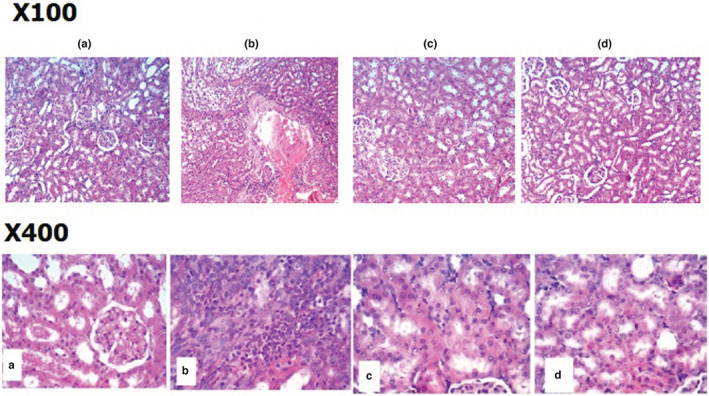
Photomicrographs of light microscopy of sections in renal tissue stained with H&E (×100, and ×400) in the four study groups. (a) Group I normal control group: showing normal kidney morphology, with no pathological changes and normal renal tubules. Normal glomerular capillary tuft and normal Bowman's space, variable‐sized tubules lined by columnar cells with eosinophilic cytoplasm and central vesicular nucleus. The interstitium shows thin‐walled blood vessels and scant stroma. (b) Group II shows marked interstitial inflammatory infiltrate formed mainly of lymphocytes with markedly congested thick‐walled vessels. Tubular epithelial cells show desquamation and hydropic degeneration with tubular necrosis, with few regenerating tubules. (c) An animal from Group III: moderate improvement in the form of decreased inflammation and many regenerating tubules with residual hydropic degeneration and very few foci of tubular necrosis. (d) An animal from Group IV: marked improvement of changes manifesting as decreased inflammation and many regenerating tubules with minimal hydropic degeneration.

As shown in Figure 2, histopathological assessment, by a pathologist blind to sample codes, was done using the renal score by Gao et al. (2014). In brief, the degree of renal tubular damage was scored as follows: 0, normal; 1, very mild focal dilatation, the area of interstitial inflammation and fibrosis, tubular atrophy, and dilation involving <25% of the field; 2, a large number of dilated tubules with the widening of the interstitium, sclerotic lesion area between 25% and 50% of the field; 3, fairly extensive dilatation of tubules and widening of the interstitium with lesions involving >50% of the field; and 4, complete atrophy of the tubules. The individual scores were then averaged for each group according to the previously described scoring system.

(b) Evaluation of the degree of fibrosis: fibrosis was determined by the area stained blue in a given field and was given a grade. In all tissue samples, 10–15 fields were analyzed for glomerular fibrosis determination.

Glomerular fibrosis was scored as follows (Sarhan et al., 2014):

1 = if fibrosis occupies <25% of 100 fields,

2 = if fibrosis occupies 25% to <50% of the 100 fields,

3 = if fibrosis occupies 50% to <75% of the 100 fields,

4 = if fibrosis occupies >75% or more of the 100 fields.

Pearson Chi‐square test was used to detect the significance between the four study groups regarding the degree of tubule/glomerular damage and fibrosis, and there was a statistically significant difference between the four groups as shown in Table 6.

**TABLE 6:** Comparison between the Pathological changes of study groups

Title		Group I control negative		Group II control positive		Group III ADMSCs		Group IV G‐CSF		*p* value#	*p* value between groups#
		No (5)	%	No (5)	%	No (5)	%	No (5)	%		
Tubular necrosis	0	5	100.00	0	0.00	0	0.00	3	60.00	0.000*	3,4 = 0.07 1,4 = 0.22 1,3 = 0.01* 2,3 = 0.02* 2,4 = 0.02*
	1	0	0.00	0	0.00	3	60.00	2	40.00		
	2	0	0.00	0	0.00	2	40.00	0	0.00		
	3	0	0.00	1	20.00	0	0.00	0	0.00		
	4	0	0.00	4	80.00	0	0.00	0	0.00		
Regenerating tubules	0	5	100.00	0	0.00	0	0.00	0	0.00	0.000*	3,4 = 0.09 1,4 = 0.01* 1,3 = 0.01* 2,3 = 0.05 2,4 = 0.07
	1	0	0.00	3	60.00	0	0.00	0	0.00		
	2	0	0.00	2	40.00	2	40.00	3	60.00		
	3	0	0.00	0	0.00	3	60.00	2	40.00		
Inflammatory cells	0	5	100.00	0	0.00	3	60.00	3	60.00	0.005*	3,4 = 0.74 1,4 = 0.22 1,3 = 0.22 2,3 = 0.01* 2,4 = 0.02*
	1	0	0.00	0	0.00	2	40.00	2	40.00		
	2	0	0.00	1	20.00	0	0.00	0	0.00		
	3	0	0.00	4	80.00	0	0.00	0	0.00		
Interstitial fibrosis	0	5	100.00	0	0.00	0	0.00	3	60.00	0.001*	3,4 = 0.10 1,4 = 0.22 1,3 = 0.01* 2,3 = 0.02* 2,4 = 0.02*
	1	0	0.00	0	0.00	4	80.00	2	40.00		
	2	0	0.00	0	0.00	1	20.00	0	0.00		
	3	0	0.00	3	60.00	0	0.00	0	0.00		
	4	0	0.00	2	40.00	0	0.00	0	0.00		

Abbreviations: ADSC, adipose tissue‐derived mesenchymal stem cells; G‐CSF, Granulocyte colony‐stimulating factor.

* Statistically significant *p* value <0.05 level, Pearson Chi‐Square test.

Both therapy groups (III and IV) showed significant improvement in tubule/glomerular damage and fibrosis compared with group II. Additionally, group IV showed better recovery of tubular necrosis and interstitial fibrosis, as shown by the insignificant difference from group I.

### 3.2 Assessment of mechanism of improvement

#### 3.2.1 Quantitative assessment of TAO and MDA in renal tissue

As shown in Table 7, Adriamycin intake leads to oxidant–antioxidant imbalance, evidenced by a significant increase in MDA and a significant decrease in TAO compared with the control negative group. There was a significant difference among all groups.

**TABLE 7:** Comparison of MDA and total antioxidants among different study groups

Title	Group I control negative	Group II control positive	Group III ADMSCs	Group IV G‐CSF	*p* value
MDA (U/mg kidney tissue)	1.18 ± 0.08	2.28 ± 0.08^a^	1.56 ± 0.21^a^ ^,^ ^b^	1.48 ± 0.19^a^ ^,^ ^b^	0.000*
Total antioxidant (U/mg kidney tissue)	71.74 ± 11.80	18.20 ± 1.68^a^	48.40 ± 8.62^a^ ^,^ ^b^	40.63 ± 11.09^a^ ^,^ ^b^	0.000*

Abbreviations: ADSC, adipose tissue‐derived mesenchymal stem cells; ANOVA, analysis of variance; G‐CSF, Granulocyte colony‐stimulating factor; MDA, malondialdehyde.

^a^ *p* value <0.05 compared with group I using post hoc Tukey test.

^b^ *p* value <0.05 compared with group II using post hoc Tukey test.

* Statistically significant *p* value <0.05 level using ANOVA test.

However, both therapy groups (III and IV) showed a significant partial improvement evidenced by a significant decrease in MDA, a significant increase in TAO compared with group II, and a significant increase in MDA and decrease of TAO in both groups compared with group I.

#### 3.2.2 Percentage changes in the CD34 ^+^ cell count

**FIGURE 3:**
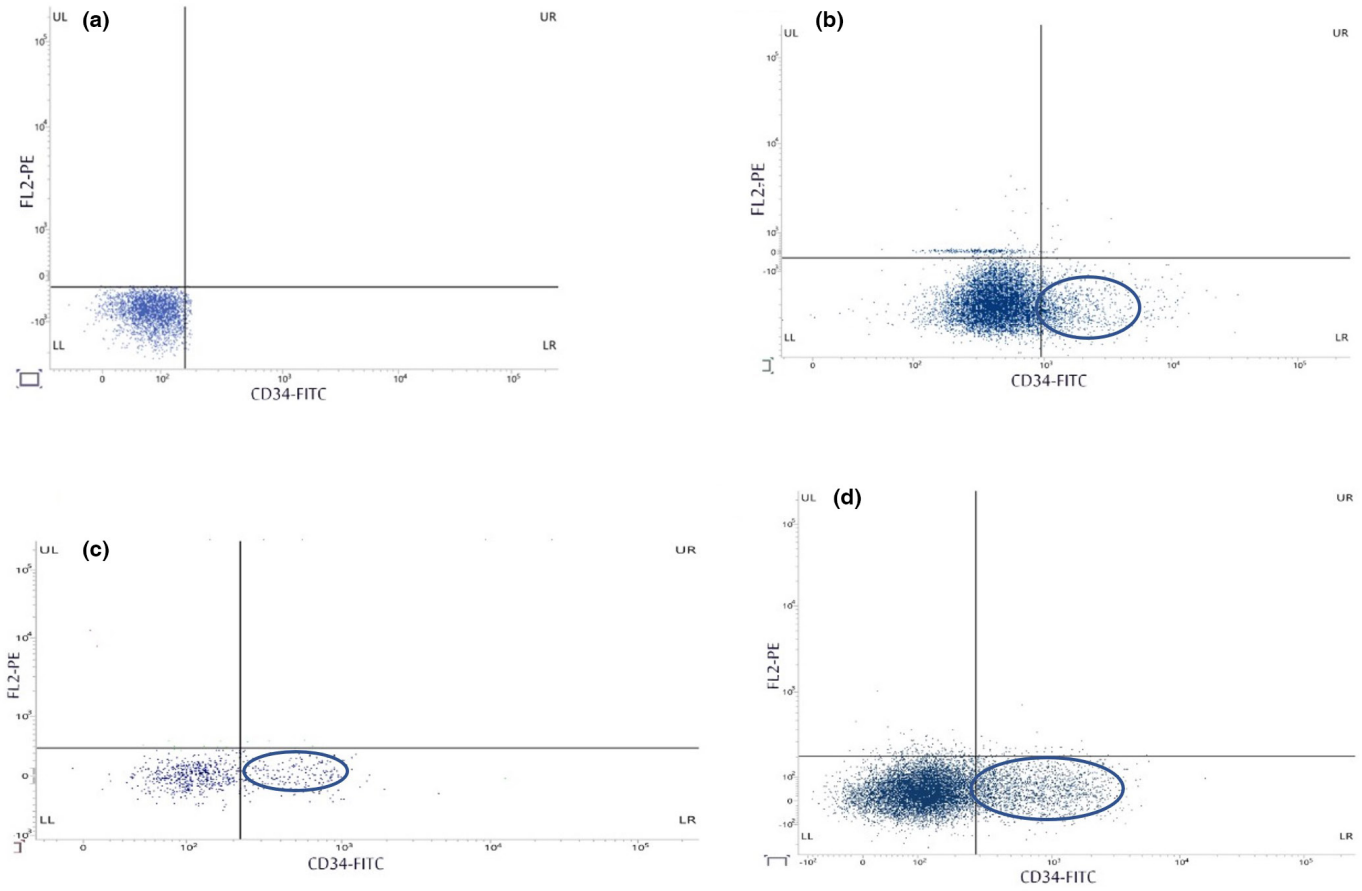
Expression of CD34‐FITC (fluorescein isothiocyanate)‐labeled cells using flow cytometry in PB. Arrows indicate positive CD34‐FITC‐labeled cells. Oval shapes indicate positive CD34‐FITC‐labeled cells; the size of which is proportional to the percentage of CD34+ mononuclear cells in the peripheral blood cells. The dot plot shows CD34 expression on the *X* axis and empty FL2 on its y axis. Accordingly, negative populations are in the lower left quadrant and positive populations for CD34 are in the lower right quadrant. (a) Group I (normal group), (b) Group II (CKD untreated group), (c) Group III (ADSC group), and (d) Group IV (G‐CSF‐treated group).

The percentage of CD34^+^ mononuclear cells measured on day 9 (Figure 3) shows a significant difference between all study groups (*p* = 0.000). Therapy Groups III and IV had a significantly higher CD34^+^ percentage than groups I and II. Group IV had a significantly higher CD34^+^ percentage than group III (*p* = 0.000; Table 8). While samples from all animals in the four groups were separately examined by flow cytometry and the results were averaged (Table 8), the results of only one representative sample of each group are shown in Figure 3.

**TABLE 8:** Comparison of the percentage of CD34+ mononuclear cells in different study groups

Title	Group I control negative	Group II control positive	Group III ADMSCs	Group IV G‐CSF	*p* value^a^
Percentage of CD34+ mononuclear cells in the peripheral blood% at day 9	0.12 ± 0.04	0.08 ± 0.02^a^ ^,^ ^c^ ^,^ ^d^	0.36 ± 0.02^a^ ^,^ ^b^ ^,^ ^d^	0.55 ± 0.11^a^ ^,^ ^b^ ^,^ ^c^	0.000*

Abbreviations: ADSC, adipose tissue‐derived mesenchymal stem cells; ANOVA, analysis of variance; G‐CSF, Granulocyte colony‐stimulating factor.

^a^ *p* value <0.05 compared with group I using post hoc Tukey test.

^b^ *p* value <0.05 compared with group II using post hoc Tukey test.

^c^ *p* value <0.05 compared with group III using post hoc Tukey test.

^d^ *p* value <0.05 compared with group IV using post hoc Tukey test.

* Statistically significant *p* value <0.05 level using ANOVA test.

#### 3.2.3 Detection of stem cell homing

**FIGURE 4:**
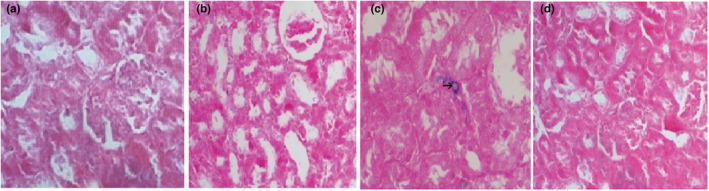
Light microscopy of renal tissue at day 84 stained with Prussian blue stain (5 mm) (×400) in the four study groups. (a) Group I normal control group shows negative staining to Prussian blue stain, (b) Group II (untreated chronic kidney disease [CKD] group) shows negative staining to Prussian blue stain, (c) Group III (CKD treated with adipose tissue‐derived mesenchymal stem cells) shows positive staining with Prussian blue, mostly peritubular and within the lining of tubules (arrow), Prussian blue stain, X400, and (d) Group IV (CKD treated with Granulocyte colony‐stimulating factor) shows negative staining to Prussian blue stain.

Homing of transplanted ADMSCs was proved by the positive stain in group III compared with negative staining of other study groups, as shown in Figure 4.

## 4 DISCUSSION

The adriamycin model is one of the classical models for the study of CKD. Adriamycin is a broad‐spectrum cell cycle, nonspecific chemotherapeutic agent. It directly damages glomerular and tubular cells via the formation of reactive oxygen species (ROS) causing glomerular sclerosis and tubular fibrosis (Fan et al., 2015). We selected this model because it reproducibly causes marked renal tissue injury after a few days of administration with a relatively low rate of mortality (<5%) and morbidity (weight loss) (LEE & Harris, 2011).

In the current study, induction of CKD was successfully established both structurally and functionally after adriamycin administration (5 mg/kg single IV injection). Structural establishment of CKD was confirmed by a significant deterioration in the tubule‐interstitial damage score of the control positive group (Adriamycin group) compared with the control negative group. Functional establishment of CKD was confirmed by a significant increase in serum urea, creatinine, and urinary protein excretion with a subsequent decrease in serum albumin and a significant decrease in HB content of Group II compared with Group I. These results are in agreement with many investigators (Fan et al., 2015; LEE & Harris, 2011; Wang et al., 2018).

In the second and third samples, on days 28 and 84, respectively, both therapy groups showed improvement in the studied kidney function parameters, including 24‐h‐proteinuria, serum albumin, creatinine, and urea levels. This improvement was not observed in the first collected samples on day 5 after administration of G‐CSF but before ADMSCs transplantation. This apparent delay might be due to the relatively low‐dose G‐CSF used in the current study. Studies that used a relatively higher dose of G‐CSF (200 μg/kg/day for three consecutive days), reported earlier functional improvement on day 3. To the best of our knowledge, none of the previous reports that used the same protocol of progenitor cell mobilization (70 μg/kg/day for five consecutive days) (Sadek et al., 2016) have examined early effects on renal regeneration after G‐CSF injection. Along the same principle, higher doses of ADMSCs (10 × 10^6^ cells) were reported to bring effects as early as the third day after induction of renal impairment(Tögel et al., 2005). In the current study, we injected ADMSCs on day 7 after adriamycin injection in order to allow sufficient time for the development of CKD.

As per the extent of improvement, our results show that the significant improvement in blood levels of urea and creatinine noted on days 28 and day 84 is partial because the values are still significantly higher than those of the control group and higher than the baseline values in the same groups. The similar findings can be noted in the 24‐h urinary protein values in the ADMSCs group, but not in the G‐CSF one, in which proteinuria did not show significant differences from the control group. Plasma albumin levels showed an apparent complete recovery in both treatment groups despite the persistent proteinuria in the ADMSCs group. This may be explained by the compensatory upregulation of albumin synthesis by the liver in situations of mild albuminuria (Tessari et al., 2006).

The noted discrepancy of the partial recovery of serum creatinine and the apparent complete improvement in proteinuria in the G‐CSF group is concordant with the recently described and still underinvestigated “nonproteinuric chronic kidney disease,” which is reported to have less renal histopathological changes and better prognosis than the classic chronic kidney disease with proteinuria (Yamanouchi et al., 2020). The histopathological results in our studies also confirm that changes in this nonproteinuric G‐CSF group are not significantly different from the normal control.

The improvement of renal functions induced, in the current study, by short‐term administration of low‐dose G‐CSF (70 μg/kg), is in agreement with (Erbas et al., 2014) and (Yan et al., 2016) who reported that G‐CSF may be a useful therapeutic agent for decreasing urinary protein excretion. Also, (Yan et al., 2016) and (Sadek et al., 2016) found significant improvement in creatinine and urea levels after G‐CSF administration. However, changing the current study's G‐CSF regimen improved the renoprotective of G‐CSF. Magen et al. (1998) showed that long‐term administration of G‐CSF for 9 years in two siblings with severe congenital neutropenia leads to proteinuria. Furthermore, (Tögel et al., 2004) showed that short‐term administration of high‐dose G‐CSF (250 μg/kg) worsened renal function.

The functional improvement in the ADMSCs group is consistent with (Huang & Yang, 2021; Sheashaa et al., 2016; Elhusseini et al., 2016; Valdés et al., 2017; Zoja et al., 2012). However, results by Sarhan et al. (2014) and Ramírez‐Bajo et al. (2020) showed nonsignificant improvement in proteinuria and creatinine levels on using bone marrow‐mesenchymal stem cells (BM‐MSCs) instead of ADMSCs. Also, (Choi et al., 2009; Magnasco et al., 2008) reported insignificant differences in urea between a BM‐MSCs‐treated group and an untreated group.

In this study, adriamycin administration to rats caused a significant decrease in Hb levels in the control positive group (Group II). In agreement with these findings (Ammar et al., 2011) found that adriamycin exposure resulted in a significant decrease in hematocrit values, thus reducing the oxygen‐carrying capacity of the blood. This can be explained by the progressive reduction of endogenous erythropoietin (EPO) levels in CKD (Portolés et al., 2021). In addition, adriamycin has an oxidizing effect on the thiols of red cell constituents, resulting in decreased glutathione stability and oxidation of hemoglobin and membrane protein, leading to the formation of large molecular weight complexes which cause hemolysis (Rocha et al., 2018).

Both therapy groups showed complete improvement in Hb levels, as evidenced by the absence of a significant difference between the control negative (GI) and therapy groups. This improvement was consistent with the findings of (Gonzaga et al., 2017) who reported that MSCs constitute an essential component of the hematopoietic niche, responsible for stimulating and enhancing the proliferation of HSCs by secreting regulatory molecules and cytokines, stimulating the BM microenvironment for hematopoiesis. Also, this improvement in Hb level was in agreement with (Nautiyal et al., 2014), who demonstrated improvement of erythropoiesis via the effect of G‐CSF. Additionally, regeneration of renal erythropoietin secreting cells can be a major cause of restoration of renal erythropoietin hormone to a normal level, stimulating erythropoiesis, which ultimately improves anemia (Rocha et al., 2018). However, (Sarhan et al., 2014) found no significant differences in Hb levels between a BM‐MSCs‐treated group and an untreated group, which disagrees with the present results. Also, (Desmond et al., 2015) showed that G‐CSF did not treat anemia. This discrepancy in results may be because they used a study population exhibiting BM failure.

Taken together, our results provide evidence of substantial but yet partial functional improvement of renal functions in the CKD rat model in response to treatment with either ADMSCs or G‐CSF.

Structurally, therapy groups (Groups III and IV) showed significant improvement in the tubulointerstitial damage score compared with Group II. This improvement was consistent with (Yong et al., 2016) who found a significant structural improvement after MSCs administration. Also, this structural improvement is in agreement with findings by Fang et al. (2010) and Li et al. (2010) who demonstrated that G‐CSF improved kidney morphological changes. However, (Magnasco et al., 2008) reported that BM‐MSCs did not alter renal damage, fibrosis, or inflammatory cell influx. Also, (Stokman et al., 2008) showed that G‐CSF did not improve structural changes. This discrepancy in results may be due to the difference in the G‐CSF dose regimen used.

In this study, the G‐CSF group showed more improvement in the tubulointerstitial damage score compared with the ADMSCs group, leading to restoration of the glomerular filtration barrier function, as evidenced by a significant reduction in protein loss in urine, which was not significantly different from the control negative value, and a consequent significant improvement in serum albumin.

Adriamycin caused an imbalance in the oxidant–antioxidant capacity manifested as a significant increase in MDA and a significant decrease in TAO capacity in the control positive (Adriamycin) group compared with the control negative group, which is in agreement with (Trypuć et al., 2018).

Both therapy groups (Groups III and IV) showed partial improvement in MDA and TAO levels as shown by the significant difference between both control positive and control negative groups. This improvement was consistent with the results of Sarhan et al. (2014) and Liu et al. (2017) who reported that MSCs had an antioxidative effect. Our results are also in agreement with those of Kojima et al. (2011) and Hortu et al. (2019) who found significant improvement in MDA and TAO levels after G‐CSF administration. Other reports suggest that MSCs have low or no antioxidant activity, such as those by Orciani et al. (2010) and Ko et al. (2012), which disagree with the present results. This discrepancy in results may be attributed to the used model, as they examined the physiological responses of MSCs to genotoxic stress via ionizing radiation. Also, in contrast to our results, (Lian et al., 2011) showed that G‐CSF increased oxidative factors, and a possible explanation for this disparate result could be that they used iron loading to induce chronic cardiac dysfunction before injecting G‐CSF to treat the mice. It is well established that chronic iron loading increases vascular oxidative stress and accelerates atherosclerosis (Day et al., 2003).

CD34 surface antigen (CD34^+^) is commonly used as a marker to identify and quantify the populations of progenitor cells (Sidney et al., 2014). In the current study, the mobilization of endogenous HSCs was enhanced by the administration of G‐CSF for five consecutive days (Aboul Fotouh et al., 2018). CD34^+^ cell count was measured in the PB using flow cytometry on the ninth day, the day of maximum HSCs mobilization (Eidenschink et al., 2012). CD34^+^ percentage was significantly elevated in both therapy groups compared with both control groups.

Concerning the ADMSCs group, this result was in line with the findings by Li and Wu (2011) who showed that MSCs stimulated and enhanced the proliferation of HSCs by secreting regulatory molecules and cytokines, providing a specialized microenvironment for controlling the process of hematopoiesis. MSCs improved the ex vivo expansion of adult human CD34^+^ PB progenitor cells and decreased their allostimulatory capacity.

In the current study, it was shown that the mobilized CD34^+^ cells in the G‐CSF‐treated group are as effective as the exogenously injected ADMSCs in reversing the histopathological and functional renal impairment compared with the untreated group. Endogenous progenitor cell mobilization from BM is a therapeutic intervention that simulates the enhanced release of progenitor cells from BM in stress situations, such as vigorous exercise, which is mediated by G‐CSF (Bonsignore et al., 2002). This intervention represents a safer and more physiological alternative to the use of exogenous allogenic or even autologous stem cell transplantation. The latter options still have the potential risks of in vitro cell manipulation, which include acquisition of the genetic and epigenetic variations while being in the cell culture (Andrews et al., 2017).

In the current study, G‐CSF and BMSC were demonstrated, to exert their reno‐therapeutic effect mainly via BM stem cell mobilization for G‐CSF and the paracrine effect on increasing CD34^+^ PB progenitor cells for the ADMSCs. In both treated groups, there was a significantly elevated percentage of CD34^+^ cells compared with the other groups. The mobilized BMSCs had migrated to the kidney to enhance damage repair. Thus, BMSCs exhibit a “homing” characteristic, whereby they migrate to the inflamed site. Helmuth (Helmuth, 2000) described this process as BMSC hearing the call of the damaged tissues. Additionally, their reported antioxidative effect can be an important factor. We have reported a significant decrease in MDA and increase in total antioxidants compared with the control positive group suggesting that stem cell mobilization via G‐CSF could exert a protective effect against ROS and preserve renal functions. These results were consistent with (Ho et al., 2012; Rochette et al., 2020)

In a recent study, G‐CSF administration was reported to have an additional direct renoprotective effect, apart from BM stem cell mobilization (Yan et al., 2020). By increasing renal infiltration of myeloid‐derived suppressor cells, G‐CSF shifts the macrophage population in the kidney toward the anti‐inflammatory M2 rather than pro‐inflammatory M1 macrophage phenotype (Yan et al., 2020). This shift in macrophage polarity was shown to be associated with the release of anti‐inflammatory mediators, growth factors, and proangiogenic cytokines that facilitate the healing process (Engel & Chade, 2019). This additional mechanism may explain the more improvement detected in proteinuria and in histopathology results in the G‐CSF‐treated group compared with ADSC‐treated one in the current study.

Administration of MSCs in this study was chosen to be on the fifth‐day postadriamycin injection (at the time of evident proteinuria) to allow sufficient time for the injury process to occur. It is known that injury is essential for adequate homing to occur. As factors released from tissue damage or apoptosis mobilize and recruit stem cells to the damaged site, ADMSCs proliferate and differentiate, eventually replacing the damaged tissues (Kojima et al., 2011).

To determine if ADMSCs homed in rat kidneys, cells were tracked in this study using the iron labeled cell technique, which demonstrated the engraftment of a few cells in the tubulointerstitial region. This result is consistent with other studies (Sarhan et al., 2014; Tögel et al., 2005). It is largely agreed that the main mechanism of the regenerative action of the stem or the progenitor cell is via their paracrine effects rather than their direct differentiation into renal parenchymal cells (Suzuki et al., 2016).

## 5 CONCLUSION

We conclude, from the current study, that mobilizing endogenous progenitor cells by G‐CSF is, at least, as effective as injecting exogenous ADMSCs in treating CKD. Actually, treatment with G‐CSF induced better improvement in histopathological findings and in proteinuria. This represents a safe definitive treatment, devoid of risks of in vitro cell manipulation. However, the dose should be optimized to try to achieve maximum structural and functional recovery.

If the reno‐therapeutic role of G‐CSF is replicable in human subjects with CKD, it can be more safe for cellular therapy, which can improve renal impairment.

## FUNDING INFORMATION

This research was fully funded by the authors.

## CONFLICT OF INTEREST

The authors declare that they have no conflict of interest to disclose.

## ETHICS APPROVAL AND CONSENT TO PARTICIPATE

All procedures performed in this study involving animals were done in accordance with the ethical standards of the Research Ethics Committee of Faculty of Medicine, Suez Canal University (Reference number: 3000#).
